# Phylogenetics of swimming behaviour in Medusozoa: the role of giant axons and their possible evolutionary origin

**DOI:** 10.1242/jeb.243382

**Published:** 2022-03-08

**Authors:** Robert W. Meech

**Affiliations:** 1 School of Physiology, Pharmacology and Neuroscience, University of Bristol, Bristol, BS8 1TD, UK

**Keywords:** Cnidarian nervous system, Escape behaviour, Fusogens, Giant axons, Nerve net, Phylogenetic tree

## Abstract

Although neural tissues in cnidarian hydroids have a nerve net structure, some cnidarian medusae contain well-defined nerve tracts. As an example, the hydrozoan medusa *Aglantha digitale* has neural feeding circuits that show an alignment and condensation, which is absent in its relatives *Aequorea victoria* and *Clytia hemisphaerica.* In some cases, neural condensations take the form of fast propagating giant axons concerned with escape or evasion. Such giant axons appear to have developed from the fusion of many, much finer units. Ribosomal DNA analysis has identified the lineage leading to giant axon-based escape swimming in *Aglantha* and other members of the Aglaura clade of trachymedusan jellyfish. The Aglaura, along with sister subclades that include species such as *Colobonema sericeum*, have the distinctive ability to perform dual swimming, i.e. to swim at either high or low speeds. However, the form of dual swimming exhibited by *Colobonema* differs both biomechanically and physiologically from that in *Aglantha* and is not giant axon based. Comparisons between the genomes of such closely related species might provide a means to determine the molecular basis of giant axon formation and other neural condensations. The molecular mechanism responsible may involve ‘fusogens’, small molecules possibly derived from viruses, which draw membranes together prior to fusion. Identifying these fusogen-based mechanisms using genome analysis may be hindered by the many changes in anatomy and physiology that followed giant axon evolution, but the genomic signal-to-noise ratio may be improved by examining the convergent evolution of giant axons in other hydrozoa, such as the subclass Siphonophora.

## Abstract (precis)

**Summary:** DNA-based phylogenetic trees chart the evolutionary origins of giant axons in the Cnidaria, a nerve net/tract condensation that may involve axonal fusion mediated by small fusogen molecules.

## Introduction

This Review explores the advantages of combining comparative genomics with comparative physiology and comparative biomechanics using closely related sister groups identified from GenBank data. It promotes the Cnidaria as a ‘model Phylum’ by concentrating on the different forms of swimming exhibited by different clades of Hydromedusae. The phylogenetic relationships between the clades, established using ribosomal DNA analysis, are associated with marked differences in their biomechanical and electrophysiological properties (Meech et al., 2021).

Prosser's (1950) analysis of comparative physiology highlighted animal preparations suitable for exploring specific areas of biology – this is often referred to as the August Krogh principle. That principle can be extended to whole phyla, and the Cnidaria, with their wide range of body forms and foraging behaviours, are ideal for the purpose. They are well represented in GenBank, with one class, the Hydrozoans, numbering over 800 different species. Cnidaria are simply constructed, with little internal anatomy and few cell types, and are ‘replete with repeated patterns of convergence’ (Cartwright and Nawrocki, 2010).

**Fig. 1.**
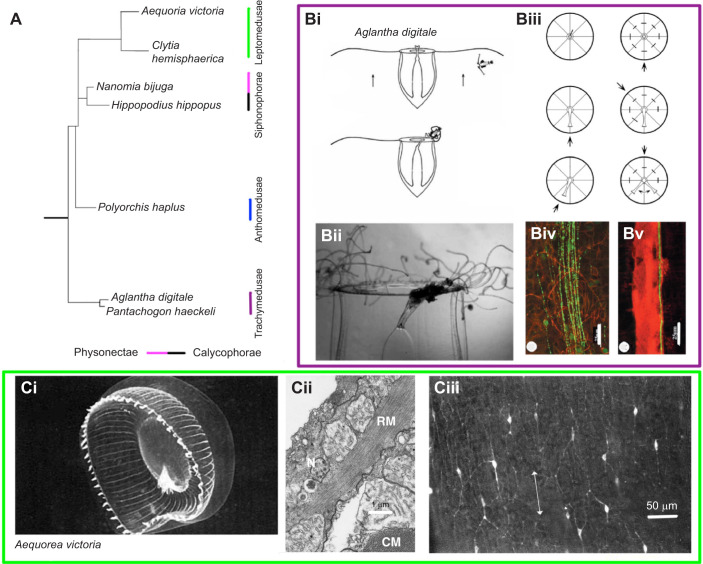
**Nerve pathways concerned with feeding.** (A) Simplified phylogenetic tree showing selected species of Trachymedusae, Anthomedusae, Leptomedusae and Siphonophorae; based on analysis of 18S and 28S nuclear ribosomal DNA and 16S mitochondrial ribosomal DNA by Cartwright and Nawrocki (2010). (Bi) Sketch of *Aglantha digitale* capturing a copepod. (Bii) Manubrium reaching over to the bell margin (‘pointing’) where tentacles hold a copepod; bell diameter about 1 cm. (Biii) Effect of cutting different small axon bundles on pointing response; eight small axon bundles are shown running from margin to manubrium; electrical stimulus used as a substitute for prey; arrow shows stimulation site; left: preparation with no cut axons, the manubrium accurately 'points' to the stimulus, suggesting that signals travel around the bell margin; right: top - preparation with all axons cut, no response to stimulus; right centre and bottom: in preparations with uncut axons, manubrium 'points' to the shortest path from the stimulus. (Biv) RFamide-like immunoreactivity (green) innervating the manubrium; scale bar: 25 µm. (Bv) Giant motor axon stained with anti-tubulin (red) and small axon bundle with RFamide-like immunoreactivity (green); scale bar: 25 µm. All images are from Mackie et al. (2003). (Ci) *Aequorea victoria*: the manubrium ‘points’ to the site of marginal curling; bell diameter, 7.5 cm (Satterlie, 1985a; reproduced with permission from John Wiley & Sons). (Cii) Ultrastructure of ectodermal muscle in the bell wall. Radial muscle (RM) shown cut longitudinally; circular swimming muscle (CM) shown in cross-section; N, neurite; the subumbrellar cavity is at the top left; scale bar: 1 µm. (Ciii) RFamide immunoreactivity in the subumbrellar myoepithelium of *Aequorea* shows a clear oral–aboral orientation (see arrow); scale bar: 50 µm. Cii and iii are from Satterlie (2008).

As is generally known, the nervous systems of many cnidarian hydroids are based on a nerve net structure. Less well known is that in many cnidarian medusae there are well-defined nerve tracts. The degree to which these tracts are developed is quite variable, however. For example, the nerve circuits concerned with feeding in the Hydrozoan *Aglantha digitale* have an alignment and condensation not seen in near-relatives *Aequorea victoria* and *Clytia hemisphaerica* (Fig. 1A; Mackie et al., 2003; Satterlie, 2008; Weissbourd et al., 2021). The aim in this Review is to explore how these differences in organisation provide opportunities to explore the environmental, morphological and genetic influences involved in neural rearrangement.

**Fig. 2.**
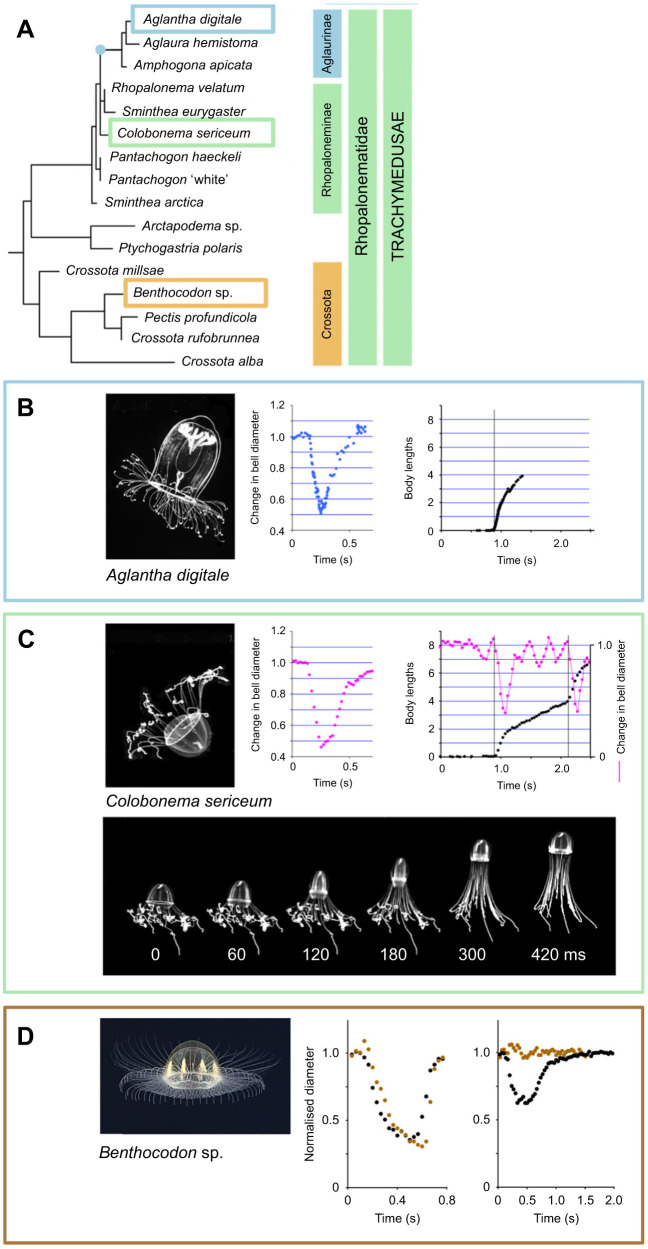
**Swimming in Rhopalonematidae.** (A) Phylogenetic relationships between four clades of Rhopalonematidae. Tree based on 18S ribosomal DNA data. The node in blue marks the appearance of giant motor axons (modified from Meech et al., 2021). (B) From left to right: *Aglantha digitale* (photograph by Claudia Mills); change in bell diameter during an escape swim; forward movement in body lengths. Initiation of the swim is shown by the vertical line (from Meech et al., 2021). (C) Top, from left to right: *Colobonema sericeum*; change in bell diameter during a fast swim; forward movement in body lengths (black; left axis). Initiation of each fast swim is shown by a vertical line; other diameter changes are slow swims. Bottom: fast swim in *Colobonema sericeum*. Video recording frame intervals are shown below. Bell refilling is incomplete even after 420 ms (modified from Meech et al., 2021). (D) From left to right: *Benthocodon* sp.; change in bell diameter during a fast swim; and during a slow swim. Brown symbols, mid-bell measurements; black symbols, bell margin measurements. Video and picture credit: Elliott et al. (2017), with permission; modified from Meech et al. (2021).

Giant axons, which have evolved multiple times in the animal kingdom, represent a highly visible outcome of a condensation process. The high conduction velocity of their action potentials, which stems from a low internal resistance, means that they are often incorporated into nerve circuits responsible for escape behaviour. The lineage leading to giant axon-based escape swimming in *Aglantha* (Fig. 2A; Meech et al., 2021) may provide a key to the developmental processes involved because the axons concerned appear to arise by fusion of multiple smaller units (Mackie, 1989; Bickell-Page and Mackie, 1991). Analysis may be aided by comparing the convergent evolution of giant axons in another hydrozoan subclass, the Siphonophorae. These colonial animals are co-ordinated by electrical impulses in a communal ‘stem’ and giant axons in some species perform roles associated with defence and tentacle management (Mackie, 1964; Mackie et al., 1987).

In plain terms, the ‘sudden’ appearance of giant axons in *Aglantha* and the other Rhopalonematidae, and again in the Siphonophorae provokes the question: can differences between the genomes of species with and without giant axons help identify the molecular basis of axonal fusion? So far as Georges Cuvier was concerned, the question is a naïve one because any change, no matter how small, would alter every other form and function of the body (Gould, 1992). Even Darwinian analysts might consider it something of a ‘long shot’ because evolutionary change is visible to us only in the occasional, somewhat distorted, ‘snapshots’ represented by extant species.

Ways to increase the chances of success are (a) to improve the resolution of the snapshot sequence by locating intermediate species; and (b) to reduce the genomic ‘noise’ obscuring precise changes by comparing species with convergently evolved characters (see Vogel, 1998). Comparative approaches of this kind are open to the attack that they are purely descriptive and without ‘any special theory or general explanation’ (Ross, 1981). To neutralise this criticism, this Review proposes a specific hypothesis, which is that giant axon generation in the Cnidaria depends on the action of small ‘fusogen’ molecules (see ‘Fusogens and giant axon formation’, below).

The arguments set out in this preamble will be discussed in more detail.

## Cnidaria as a ‘model’ phylum

The Cnidaria are a diverse collection of life forms, ranging from sessile hydroids, sea anemones and corals to highly motile Medusozoa, and questions about their being lumped together in the same phylum are not unreasonable. That they are so collected was initially due to the comparative studies of Thomas Henry Huxley (1849). Although Huxley himself acknowledged ‘the establishment of affinities among animals has been so often a mere exercise of the imagination’, his conclusions concerning affinities among the medusae have stood the test of time. They include: (1) ‘That a Medusa consists essentially of two membranes inclosing a variously shaped cavity’, and (2) ‘That the peculiar organs called thread-cells are universally present’.

His ‘foundation membranes’ we recognise as epithelia, and his ‘thread cells’ are what we call cnidae.

A simple two-layer structure with the same few components being adapted to a range of different environmental niches may explain why the phylum contains so many examples of convergent evolution (Cartwright and Nawrocki, 2010). It is this very simplicity that makes the Cnidaria a ‘model phylum’ for studying the cellular basis of behaviour.

## Advantages of the hydrozoa

Phylogenetic studies suggest that the ancestral cnidarian was a solitary polyp (Kayal et al., 2018). That polyp gave rise to three clades: the Anthozoa, which includes sea anemones and different forms of coral, the parasitic Myxozoa and the motile Medusozoa. The Medusozoa, in turn, are made up of four classes of jellyfish: the Scyphozoa, the Cubozoa, the Staurozoa, and the Hydrozoa. Behaviourally the cubozoans, or box jellyfish, are probably the most complex, but the largest of the medusozoan classes, and the most diverse, is the Hydrozoa (Collins, 2009) and for this reason, they are the focus of this Review.

## Bell-based swimming

The functioning of the nerve circuits responsible for different forms of swimming in the Hydrozoa can only be fully appreciated in the context of their bell mechanics. The motive force for swimming arises in the cellular layer, identified by Huxley as a ‘foundation membrane’, on the underside of the swimming bell. In what we now call the subumbrella epithelium, each cell body is attached to one, sometimes two, striated muscle tails. In Hydrozoa, these muscle epithelial cells are connected by gap junctions through which electrical currents pass freely (Mackie and Singla, 1975; Kerfoot et al., 1985). ‘At each contraction of this muscular sheet the gelatinous walls of the bell are drawn together; the capacity of the bell being thus diminished, water is ejected from the open mouth backwards and the consequent reaction propels the animals forwards. In these swimming movements, systole and diastole follow one other with as perfect a rhythm as they do in the beating of a heart’ (Romanes, 1898). The ‘gelatinous’ wall of the bell, now called the mesogloea, has elastic properties (DeMont and Gosline, 1988). It flexes when the swimming muscles contract (‘systole’) and springs back into shape (‘diastole’) when they relax.

**Fig. 3.**
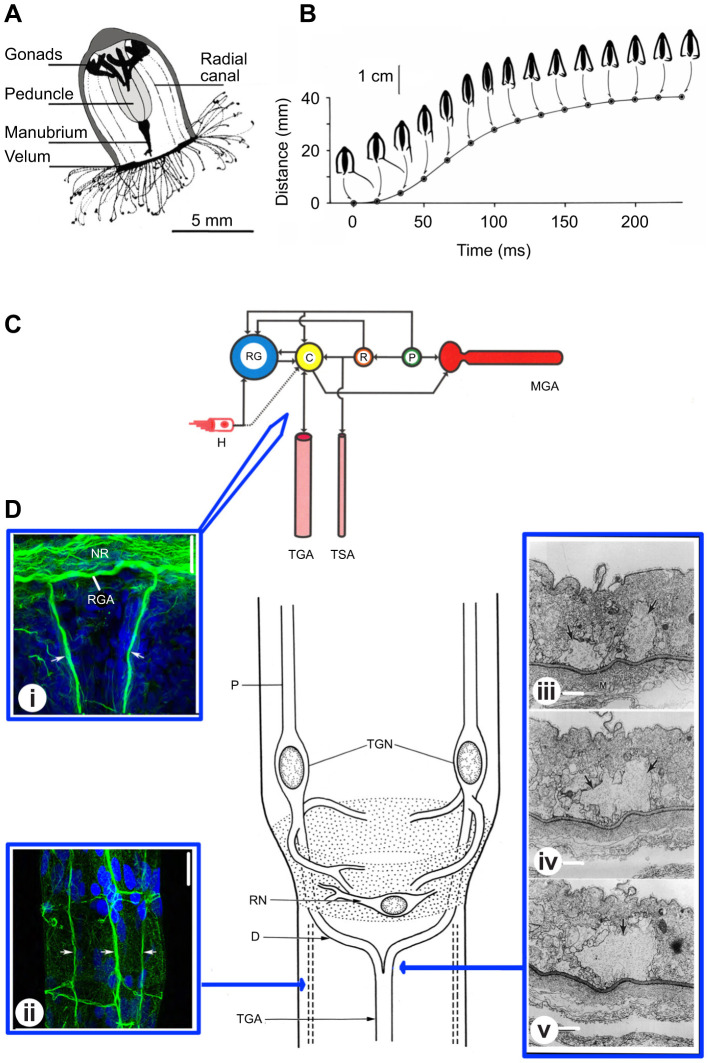
**Tentacle giant axons in escape swimming in *Aglantha digitale*.** (A) *Aglantha digitale* section showing general anatomy. (B) Escape swim; profiles showing bell shape changes and position, based on stroboscopic data, combined with cinematographic data on tentacle withdrawal (from Donaldson et al., 1980; ^©^Canadian Science Publishing or its licensors). (C) Escape swim nerve circuitry is presented as a section through the nerve ring, level with one of the giant axons – connections between motor giant axon (MGA), pacemaker (P), relay (R) and carrier (C) systems, and the ring giant axon (RG) are based on electrophysiological data. The ring giant/carrier system receives an input from numerous hair cells (H) around the bell margin. Outputs to the tentacles, i.e. the tentacle giant axon (TGA) and slower conducting tentacle axons (TSA) are also shown (from Mackie and Meech, 1995). (D) Centre: Sketch of tentacle giant neurons (TGN), their proximal (P) and distal neurites (D). The tentacle giant axon (TGA) is formed from two distal neurites. Also shown is one of a number of ring neurones (RN); position of tentacle slow axons shown by dashed line (modified from Bickell-Page and Mackie, 1991; reproduced with permission from the Royal Society, UK). (i) Proximal neurites (arrows) of tentacle giant neurons may make contact with the ring giant axon (RGA)/carrier system in the nerve ring (NR); α-tubulin immunoreactivity (green); scale bar, 30 µm (from Norekian and Moroz, 2020; reproduced with permission from John Wiley & Sons). (ii) Processes arising in the region of the ring neurons travel distally (arrows). The central process is thicker than the other two and may correspond to the tentacle giant axon. The lateral processes may correspond to the tentacle slow axons; α-tubulin immunoreactivity (green); scale bar, 20 µm (from Norekian and Moroz, 2020; reproduced with permission from John Wiley & Sons). (iii–v) Electron micrographs of 1 µm cross-sections through distal neurites from two tentacle giant neurons showing that they fuse to form the tentacle giant axon (arrows); scale bars, 2 µm (from Bickell-Page and Mackie, 1991; reproduced with permission from the Royal Society, UK).

In a comprehensive description of medusan swimming, Gladfelter (1972, 1973) reported that most hydromedusae swimming velocities were in the range 2.5–9 cm s^−1^, but he picked out *Aglantha* (Fig. 3A) as of particular note because its powerful contraction might provide the means to escape from a predator. During an escape swim, a single contraction propels *Aglantha* by five body lengths with a maximum velocity of about 40 cm s^−1^ (Fig. 3B; Donaldson et al., 1980; Meech, 2015).

According to Gladfelter (1973), the hydrozoan swimming system ‘has a number of structural parameters’, including bell shape and mesogleal consistency, ‘which can vary through a whole spectrum of possibilities’. Functional parameters include maximum and average velocities, acceleration and turning radius. To this list might be added the drag generated by the tentacles and their management by the nervous system (see ‘Tentacle management’, below).

Gladfelter (1972) drew attention to the role of the velum, which is a muscular shelf around the opening at the base of the bell (Fig. 3A). During each swim the velar circular muscles contract, constricting the bell aperture and increasing thrust by increasing the velocity of the expelled water. The velum also has a layer of radially aligned muscles whose local contractions can displace the aperture so that the water is expelled obliquely, causing the animal to turn.

In broader shaped hydromedusae, Gladfelter (1973) found that although the velum may still act as a nozzle, its contribution to turning is relatively small and asymmetrical contractions at the bell margin are more important. In these jellyfish a combination of the velum and bell margin acting together produce a smaller turning radius than velar action alone.

## Mechanics of swimming in medusae – jetting and rowing

For many years, jet propulsion was thought to be the principal thrust-generating mechanism in jellyfish (DeMont and Gosline, 1988). However, Colin and Costello (2002) showed that while this was true for species where the bell height is greater than the bell diameter (variously described as prolate, bullet- or thimble-shaped), acceleration patterns in flatter, more disc-shaped forms could not be modelled by assuming jet propulsion alone. Swimming contractions in these oblate jellyfish were limited to the bell margin, and the wake produced acted more like a paddle (Ford and Costello, 2000). Jet propulsion does contribute to thrust even here, but its importance depends upon the shape of the bell and its contraction characteristics (Colin and Costello, 2002).

Many species’ foraging strategies appear to be set by the shape of the bell and its contraction characteristics. During paddle swimming, a large amount of water is moved slowly and economically. Energy consumption, which is related to the square of the water velocity (Vogel, 1994), is low and so swimming can be continuous. What is more, the bell contractions can form vortex rings, which bring food particles into contact with trailing tentacles (Costello and Colin, 1994, 1995). In contrast, jet propelled swimming is a much more energy-consuming process because water is expelled at such a high velocity. Jet swimmers spend much of their time passively hanging in the water poised to trap their prey. Swimming is reserved for escape or to gain height in the water column.

## Predator evasion – crumpling and jetting

A comparative study by Hyman (1940) showed that the exumbrellar surface of most Hydromedusae, although insensitive to light touches, has a protective response to a heavier mechanical stimulus that Hyman called ‘crumpling’ – ‘the animal ceases pulsations, folds in the bell to the smallest possible compass, and sinks’. The effect is to protect the gonads, tentacles, mouth and key parts of the nervous system (Mackie and Passano, 1968). Crumpling is produced by contractions in narrow bands of radial muscle fibres that run over the subumbrella surface at right angles to the myoepithelial cells. *Aequorea victoria* appears to be a special case: its radial fibres cover the whole of the subumbrella (Satterlie and Spencer, 1983; Satterlie, 2008) but ‘it gives no crumpling response even to severe blows’ (Hyman, 1940). *Aequorea* does give a restricted local contraction, however. This is called a ‘radial response’ (Satterlie, 1985a,b) to distinguish it from more generalised crumpling.

**Fig. 4.**
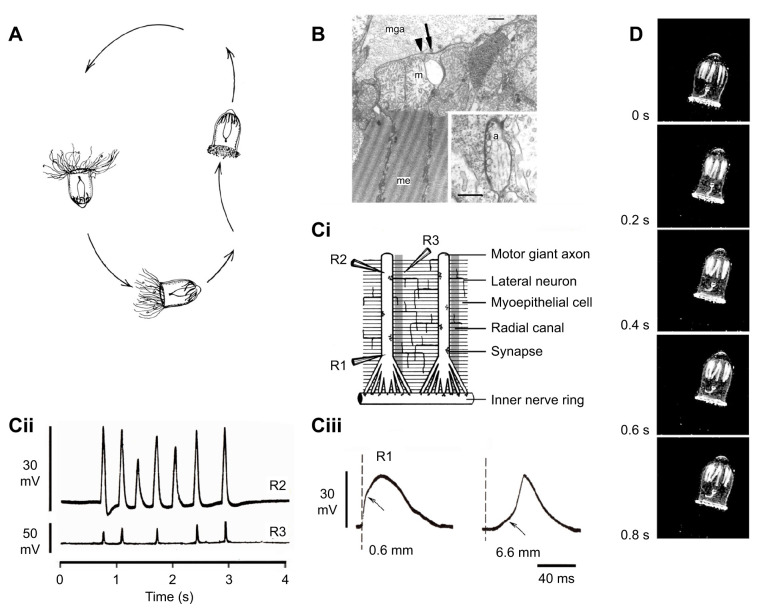
**Slow swimming in *Aglantha digitale*.** (A) Drawing of foraging behaviour showing the pattern of intermittent slow swimming (from Mackie, 1980; ^©^Canadian Science Publishing or its licensors). (B) Electron micrograph of a cross-section of the bell margin showing a motor giant axon (mga) lying over the swimming muscle epithelium (me) made up of electrically coupled myoepithelial cells, with large mitochondria (m), and striated muscle tails; a gap junction (arrowhead) lies close to a synapse containing vesicles (arrow); scale bar: 1 μm. Inset, bottom right, shows a cross-section through a lateral nerve axon synapse (a) onto the muscle epithelium; inset scale bar: 0.5 μm (from Kerfoot et al., 1985). (Ci) Diagram of the nerve circuitry responsible for slow swimming. Two (of eight) giant motor axons are shown lying over the sheet of electrically coupled myoepithelial cells. Axon and epithelium are connected by chemical synapses. A lattice of lateral neurons is electrically coupled to the motor giant axon and connected with the myoepithelium by chemical synapses. Lying alongside each giant axon is a radial canal, part of the digestive system. Recording sites R1 and R2 are in the axon; R3 is in the muscle epithelium about 80 μm from R2. (Cii) Intracellular records during a sequence of slow swims; upper trace, from giant motor axon 5.5 mm from the nerve ring; lower trace, from the nearby myoepithelium. Mg^2+^ (65 mmol l^−1^) in seawater was used to reduce muscle contraction while retaining synaptic transmission (from Meech and Mackie, 1993). (Ciii) Slow swim spike electrogenesis in giant motor axon at different distances from the bell margin. Arrow shows the changing amplitude of the synaptic potential. Axonal resting potential was −66 mV (from Meech and Mackie, 1995). (D) Video frames of a slow swim captured at 1/300 s; 0.2 s intervals; the bell height about 2 cm (from Meech, 2015).

At the other extreme is *Aglantha digitale*. Instead of crumpling, *Aglantha* has a strong reflexive escape swim, during which it can attain a velocity of 40 cm s^−1^ (Donaldson et al., 1980; Meech, 2015). The reflex is initiated by ‘hair cell’-like vibration receptors distributed around the rim of the bell (Fig. 3C; Arkett et al., 1988). When stimulated, they excite action potentials in a giant ring axon that inputs onto each of the eight giant motor axons that run up the bell beside the eight radial canals (Fig. 3A; Fig. 4Ci).

## Nerve tracts versus nerve net

Fig. 1A shows the phylogenetic relationship between the species referred to in this section, *Aglantha*, *Clytia*, *Aequorea* and *Polyorchis*, as well as others discussed in this Review.

### Feeding in *Aglantha*, *Clytia* and *Aequorea*

Feeding poses something of a problem for all Cnidaria; in many species the manubrium holds a central position while the tentacles trap food out on the periphery. In medusae with a narrow bell, like *Aglantha*, the long pendant manubrium can reach over and collect the prey from the base of the contracted tentacle (Fig. 1Bi,ii; Mackie et al., 2003). However, for *Aequorea* with its broad flat bell and short manubrium, the bell margin must fold inwards and bring the trapped prey all the way to meet the manubrium (Fig. 1Ci; Satterlie, 1985a). For *Aglantha* the challenge is for the mouth to find the food; for *Aequorea* the margin with its trapped food must find the mouth.

In *Aequorea* the area of muscle involved with transferring food covers a number of interradial segments (Fig. 1Ci). The radial muscles responsible are innervated by a nerve network that stains for RFamide (Fig. 1Cii,iii). In *Clytia*, which resembles *Aequorea* in its food transfer technique, the nerve net also stains for RFamide. It is functionally organised into ‘wedge-shaped zones’ that stretch from the margin to the mouth (Weissbourd et al., 2021). A flexible local connectivity between the zones may increase the precision of food transfer.

In *Aglantha* the manubrium can locate the trapped food with a high degree of accuracy. Unlike *Aequorea* and *Clytia*, *Aglantha* has no nerve net and no subumbrella radial muscle. Instead, messages travel around the bell margin in the bundle of axons that make up the nerve ring, and then up to the manubrium via eight small RFamide-staining nerve tracts running beside each radial canal (Fig. 1Biii,iv,v). We suppose that precision is achieved in food transfer in part by summation of electrical events in the manubrium itself. The conduction velocity of the first impulse to arrive (15 cm s^−1^) is faster than the second (11 cm s^−1^). Consequently, the further the impulses travel, the more separated they become and the weaker the summated contraction in the manubrium (Mackie et al., 2003).

The condensed system of nerve tracts in *Aglantha* might confer an advantage based on the lower energy consumption of the small amount of muscle involved. However neural condensation is just one of a suite of changes, some of which involve the animal's foraging behaviour.

### Swimming in *Aglantha*, *Polyorchis* and *Aequorea*

The swimming pattern described by Romanes (1898) as resembling a beating heart depends for its rhythmicity on pacemaker neurons in the nerve ring at the base of the swimming bell. As described by Satterlie (1985a): ‘The “basic” hydrozoan swimming system consists of an electrically coupled network of neurons, found in the inner nerve ring, which synaptically activates an overlying sheet of electrically coupled epithelial cells’. In different hydrozoan species there are a variety of links between the endogenous pacemaker and the swimming musculature. As with feeding, some species are more nerve net based; others rely on nerve tracts. In *Aequorea* the spread of excitation depends on neural activity in a widely distributed subumbrellar nerve net aided by current spread through electrically coupled epithelial cells. In *Aglantha*, the nerve net is absent and a set of eight giant axons and a lattice of well-defined lateral neurons take its place (Roberts and Mackie, 1980; Kerfoot et al., 1985; Fig. 4Ci). In *Polyorchis* the nerve tracts are less well developed; the bell is divided into quadrants with innervation at the periphery of each quadrant and the spread of excitation depending on impulses generated within the muscle itself (Spencer, 1978, 1979).

The advantage of a nerve tract solution is seen most clearly in *Aglantha*, where giant axons transmit excitation rapidly from margin to apex of the bell. There are also benefits derived from the increase in signalling bandwidth (see ‘Slow swimming in *Aglantha* and *Colobonema*’, below). *Aequorea*’s nerve net solution, however, has the advantage of flexibility. ‘*Aequorea* can “turn off” portions of the swimming system while the remainder of the bell undergoes apparently normal swimming contractions’ (Satterlie, 1985a). Moreover, in *Aequorea* each bell contraction is associated with a burst of action potentials in the swim motor network (Anderson and Mackie, 1977; Satterlie and Spencer, 1983), which provides for more flexibility in adjusting righting movements (see ‘Slow swimming in *Aglantha* and *Colobonema*’, below).

### Inhibitory input to swim pacemakers

In *Aequorea*, swimming-generated water currents provide a continuous supply of food to the tentacles. Nevertheless, these swimming movements may decrease the likelihood of a successful transfer of food from the tentacles to the manubrium. Local swimming inhibition (Satterlie, 2008) would promote transfer while allowing the rest of the bell to maintain its swimming rhythm so as to keep up the food supply. Jet swimming movements in *Aglantha* would also displace food during transfer, but to have any effect swimming must be inhibited more generally. Contact of the manubrium by food stimulates impulses within a plexus of nerves associated with each of the eight radial canals. At the margin each nerve plexus synapses with pacemaker cells and the nerve impulses produce prolonged inhibitory potentials there (Mackie et al., 2012). The effect is to inhibit the entire pacemaker system, presumably because the inhibition occurs at all eight points around the bell margin.

## Giant axons in predator evasion

The most striking aspect of *Aglantha*’s behaviour is the power of its escape swim. The motor axons responsible have a 40 µm diameter (Fig. 4B), which justifies the use of the term ‘giant’ and reduces their longitudinal resistance so that their action potentials can travel at high speed (1–4 m s^−1^ at 10°C; Roberts and Mackie, 1980; Mackie and Meech, 1985). The transfer of excitation from margin to apex of the bell is so short as to be effectively instantaneous, compared with the time course of contraction. Intracellular records from either side of the neuromuscular junction show that transmission from axon to muscle is also fast (synaptic delay 0.7 ms at 10°C; Kerfoot et al., 1985). The uniform contraction of the bell maximises the thrust because a ‘nozzle’ forms at the bell margin, causing the expelled water to exit at high velocity.

The strength of *Aglantha*’s fast swim is unusual and eye-catching, but some closely related jellyfish also have strong swims (Mills et al., 1985, 1996). All these jellyfish species belong to the Order Trachymedusae, specifically to the Family Rhopalonematidae. Film captured for the BBC Blue Planet II series showed moreover that one member of the family, *Colobonema sericeum*, could perform both fast and slow swims just like *Aglantha* (Fig. 2C). *Colobonema* lives at around 400 m in the depths of the temperate Pacific Ocean but Steven Haddock and members of the Monterey Bay Aquarium Research Institute staff on board R/V* Rachel Carson* were able use the ROV *Vantana* to capture several specimens and bring them to the surface. Here they survived for several days maintained in seawater at 6°C. They were vibration sensitive and had particularly fast and strong swims (comparable to *Aglantha*; Fig. 2B,C), even though no giant axons were visible in their body wall (Meech et al., 2021).

Fig. 2A shows that the Rhopalonematidae consist of four subclades and in only one of them, the Aglaura subclade, is there any evidence that giant axons play a role in swimming. It appears that giant axons were introduced into the Aglaurae by the lineage leading from the Rhopaloneminae (which includes *Colobonema*). How does *Colobonema* generate such rapid fast swims without the benefit of giant motor axons? One explanation is that the myoepithelium is itself regeneratively active, as it is in *Polyorchis* (Spencer, 1978), and its unusually large thickness provides a low internal resistance. Local circuit theory (see Hodgkin, 1954) suggests that the lower the internal resistance, the higher the rate at which depolarising current discharges the membrane capacity, and hence the higher the rate of conduction.

## Slow swimming in *Aglantha* and *Colobonema*

When foraging, *Aglantha* sinks in the water column with its tentacles extended ready to trap prey (Fig. 4A); a series of slow swims then returns the animal to the top of its cycle (Mackie, 1980). In this mode the giant motor axon (Fig. 4B,Ci) generates sequences of low amplitude spikes (Fig. 4Cii, top), leading to depolarising events in the muscle (Fig. 4Cii, bottom). Muscle contractions are limited to the mid-bell region (Fig. 4D), the advantage being that the bell orifice remains wide open and the expelled water travels at a low rate. Slow swims are therefore energy conserving (Vogel, 1994).

The contractile element in the bell is a muscle epithelium electrically coupled throughout with gap junctions (Fig. 4Ci). Differences in the strength of contraction occur at different locations because the muscle response depends on the rate of rise of the axonal event (R.W.M. and G. O. Mackie, in preparation). The shape of this event changes as it travels from the margin towards the apex of the bell (Fig. 4Ciii). At the margin it is dominated by a simple synaptic potential, arising from pacemaker synapses in the nerve ring. This component (Fig. 4Ciii, arrow) decrements exponentially as it spreads along the axon, leaving the slow swim spike to propagate up the axon increasing in size as it does so. The electrical response in the muscle increases along with the increase in the axonal spike component.

*Aglantha* giant axons are remarkable for having double the signalling bandwidth of other axons. They transmit two kinds of signal: rapidly propagating high threshold action potentials and slower, low amplitude, low threshold ‘spikes’ (Mackie and Meech, 1985). The peak of the slow swim spike remains below the threshold of the escape swim action potential. Although some jellyfish swim with different ‘gaits’ (Megill, 2002), *Aglantha*’s ability to switch between slow and fast swimming is remarkable. This is not only because of the strength of its fast swims but also because the two forms depend on two different neural circuits (Singla, 1978): fast swimming is linked to vibration receptors in the bell margin whereas slow swimming is driven by pacemaker neurons in the nerve ring (see ‘Nerve tracts versus nerve net’, above).

Slow swimming in *Colobonema* differs from that in *Aglantha* because in *Aglantha* it is the mid-bell region that contracts, whereas in *Colobonema* (and members of the Crossota subclade; Fig. 2D) contraction is restricted to the bell margin. Along with the difference in mechanics go differences in neural control, which gives *Colobonema* flexibility in adjusting its righting movements (Meech et al., 2021).

## Fusogens and giant axon formation

Giant axons appear at multiple sites in the phylogenetic tree; they are found in the Cnidaria, Platyhelminths, Nemertines, Phoronids, Hemichordates, Chordates, Molluscs, Annelids and Arthropods (Bullock, 1984). Young (1936) estimated that in the squid *Loligo forbesii* they arise from the fused processes of 300–1500 separate nerve cell bodies. There are similar formations in earthworm giant axons (Stough, 1926). In *Aglantha* the motor giant axons are multinucleate and ‘not infrequently show bifurcations or give off side branches which flow back into the main axon after wandering separately for some distance’ (Mackie, 1989). In some cnidarians there is evidence of internalised cell membranes having arisen by one neurite engulfing another (Mackie et al., 1988; Spencer, 1979) and in *Aglantha* the tentacle giant axon arises from the neurites of two giant neurons (Bickell-Page and Mackie, 1991; Fig. 3D). Identification of the phylogenetic branch leading to giant axon-based escape swimming in *Aglantha* (Meech et al., 2021) may provide a key to analysing this fusion process.

It is possible that axonal fusion is promoted by small intracellular proteins (fusogens) that, by a variety of means, draw membrane surfaces together (Segev et al., 2018). If so, fusogens are likely to be expressed in early development. Giant axons are absent from the bells of early post-larval specimens of *Aglantha*, but they are well formed in animals with bells larger than about 1 mm diameter (Mackie, 1989). Possible pre-giant axon neuroblasts are aligned along the radial canals (Mackie, 1989) and transcriptomes from them might help establish whether fusogen molecules play a role. Other approaches are considered in the Discussion.

## Role of giant axons in siphonophores

The relative simplicity of the Cnidaria suggests that convergently evolved characters might develop by mediation of the same molecular toolkit. If so, comparing the genomes of species with convergently evolved characters could be a way to identify their common molecular basis.

**Fig. 5.**
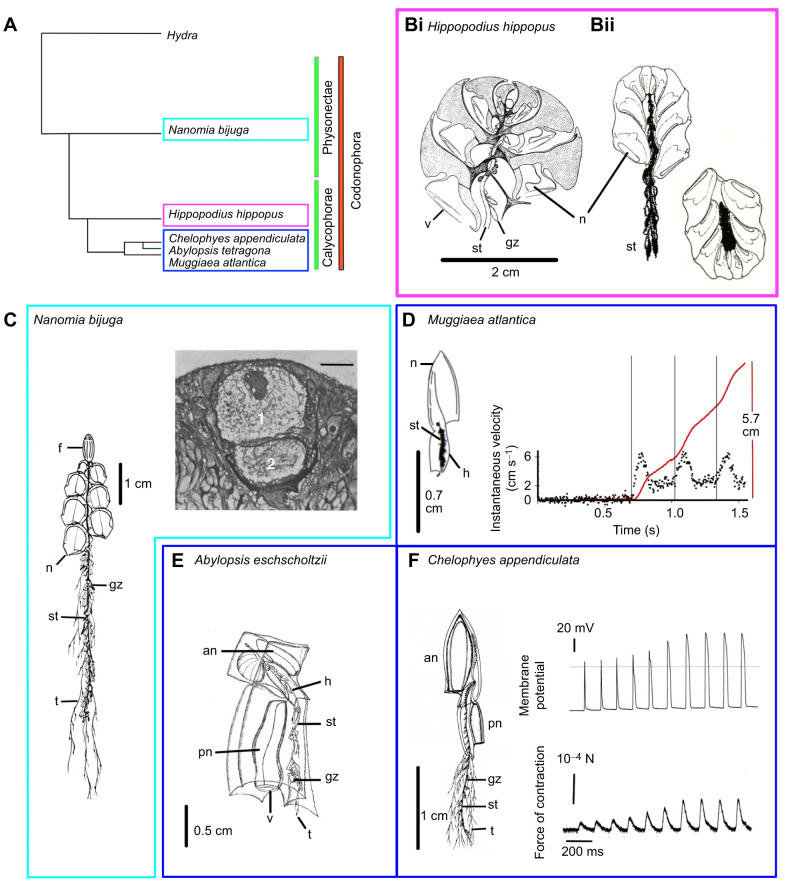
**Swimming in codonophoran siphonophores.** (A) Simplified phylogenetic tree showing relationships between five codonophoran siphonophores; based on analysis of 18S nuclear ribosomal DNA and 16S mitochondrial ribosomal DNA by Dunn et al. (2005). (B) *Hippopodius hippopus.* (i) Line drawing showing multiple nectophores (n) and truncated stem (st) (adapted from Chun, 1897). (ii) Change in orientation caused by a shift in center of gravity after contraction of stem (st) and tentacles (from Jacobs, 1937; reproduced with permission from Springer Nature). (C) *Nanomia bijuga*; left: line drawing of a young specimen showing stem (st), tentacles (t) and multiple nectophores (n); it is kept upright by a float (f) (from Mackie, 1963; reproduced with permission); right: 1 μm Epon cross-section of the stem showing two giant axons (‘1’ and ‘2’); note nucleus in top axon. Scale bar: 10 μm (from Mackie, 1973; reproduced with permission from Seto Marine Biological Laboratory, Kyoto University). (D) *Muggiaea atlantica*; left – sketch showing single nectophore (n) with stem (st) fully retracted into the hydroecium (h); right – first swims in a sequence showing instantaneous velocity (left axis; data points, 12.5 ms rolling average) and change in position (right axis, red line; vertical lines mark start of swims); specimen filmed at 240 frames s^−1^ at 11°C; stem only partially retracted (R.W.M., unpublished). (E) *Abylopsis eschscholtzii*; line drawing showing a small cuboidal anterior nectophore (an) and larger posterior nectophore (pn). The stem (st) and most of the tentacles (t) are drawn into the hydroecium (h; adapted from Chun, 1897). (F) *Chelophyes appendiculata*; left – line drawing showing large anterior nectophore (an); smaller posterior nectophore (pn); the trailing stem (st) and tentacles (t) have been truncated (adapted from Mackie, 1984; reproduced with permission from Springer Nature); right – upper trace, intracellular recording from muscle cell during a swim series; dotted line shows 0 mV; scale bar: 20 mV. Lower trace, strain gauge record of the force of contraction; scale bar: 10^−4^ N; time scale 200 ms (from Inoue et al., 2005). All five siphonophores have multiple gastrozooids (gz): the nectophore velum (v) is not always visible.

Giant axons evolved in the Aglura subclade of the Rhopalonematidae and again in the Siphonophorae. The phylogenetic relationship between the physonect and calycophoran orders of the Siphonophorae, discussed here, is shown in Fig. 5A, based on analysis of ribosomal DNA by Dunn et al. (2005). *Chelophyes appendiculata* is a small calycophoran with two linked swimming bells (nectophores) and a trailing stem (Fig. 5F). Like *Aglantha*, *Chelophyes* can perform a type of dual swimming. When foraging, it maintains its position in the water column by occasional contractions of its smaller posterior nectophore. ‘When the colony is disturbed, however, it shoots away with astonishing rapidity, attaining instantaneous velocities up to 30 cm/s during short bursts of contractions of both anterior and posterior nectophores … During such escape movements, the stem is retracted to minimize drag and increase escape speed’ (Bone and Trueman, 1982).

Siphonophores are colonial animals and for coordination they rely on neural impulses in the stem, the structure that links the different parts of the colony together. In *Chelophyes*, the stem houses two 10 µm diameter giant axons that innervate its muscle epithelium (Mackie and Carré, 1983). The rapidity of stem retraction is attributable to the high speed of axonal conduction. A similar axon runs to the anterior swimming bell and provides a fast pathway for excitation of its subumbrella swimming muscles (Mackie and Carré, 1983).

*Muggiaea atlantica* is another calycophoran closely related to *Chelophyes* (Fig. 5A), but its posterior swimming bell is undeveloped and so dual swimming, of the kind exhibited by *Chelophyes*, is not possible. The stem does, however, contain two large axons (7 µm diameter; Satterlie and Spencer, 1983). Local disturbances in the water cause *Muggiaea* to retract its tentacles, although not its stem. Once the tentacles have been withdrawn swimming begins, each contraction driving the animal forward an equal distance (Fig. 5D) with an instantaneous velocity reaching 6 cm s^−1^. This is a similar velocity to *Chelophyes* when swimming with the small posterior nectophore alone (Bone and Trueman, 1982). In another near relative, *Abylopsis* sp., it is the anterior nectophore that is much reduced (Fig. 5E). *Abylopsis* also swims at a single speed and its instantaneous velocity reaches 8 cm s^−1^ (Bone and Trueman, 1982).

*Chelophyes* and *Muggiaea* both have giant axons, but a third slightly more distantly related calycophoran, *Hippopodius hippopus* (Fig. 5A), does not. It has multiple swimming bells, which are arranged in such a way as to provide a central space into which the stem can be withdrawn when the animal is touched or stimulated (Fig. 5B; Jacobs, 1937). *Hippopodius* exhibits no concerted locomotory movements and when stimulated individual nectophores operate independently (Mackie and Boag, 1963). ‘When disturbed the stem contracts strongly and the appendages are lifted into a position between the swimming bells. Thus, the center of gravity is shifted to the top of the colony and the whole body turns over [Fig. 5Bii, right]. Since in this new orientation the swimming bells expel water upward, the colony is actually able to propel itself downward’ (Jacobs, 1937, 1962).

*Hippopodius* feeds exclusively on ostracods (Purcell, 1981), which are relatively slow moving compared with copepods, the natural prey of *Muggiaea* and *Chelophyes*. Copepods are quite capable of damaging soft tissues if trapped within the swimming bell – hence the need for some mechanism, like fast swimming, to flush out strays. In place of fast swimming, *Hippopodius* has evolved a number of other defensive behaviours, which include involution, bleaching and luminescence (Bassot et al., 1978). It is not clear whether the *Hippopodius* lineage lost the ability to generate giant axons or whether they evolved separately in the physonects and calycophorans.

The physonectid order of siphonophores can be distinguished from their calycophoran cousins (Fig. 5A) by the gas-filled float that keeps them upright in the water column (Fig. 5C). In *Nanomia bijuga* giant axons convey impulses rapidly to a set of 10–20 nectophores, where they produce concerted bell contractions and generate a vigorous swim. Swim velocities are 20–30 cm s^−1^ compared with 8–10 cm s^−1^ during normal swimming (Mackie, 1986). Giant axons also excite the stem to contract either locally or along their entire length (Mackie, 1971). In *Nanomia* they may be 30 µm in diameter and have multiple elongated nuclei (Fig. 5C). One may think of them ‘as thickened, condensed, longitudinally orientated portions of the diffuse nerve net’ (Mackie, 1971). Other physonects like *Forskalia* and *Halistemma* also have giant axons in their stem (Grimmelikhuijzen et al., 1986).

## Tentacle management

The problem that Hydrozoans have of getting food from the bell periphery into the more centrally located mouth is discussed in ‘Nerve tracts versus nerve net’ (see above). The siphonophores have resolved the problem in their unique fashion by having multiple mouths. It means that the tentacles are no longer clustered around a single bell, but can be deployed in an elaborate net, trapping food over a large volume of ocean. One consequence is that food transfer occurs via random movements of the mouth. Another is that tentacle management is especially important because the more extensive the net, the greater the drag generated during swimming.

Comparisons between species show that there are broadly two ways to solve the drag problem. *Muggiaea* withdraws its tentacles before it starts to swim; in *Chelophyes*, swimming sequences start before the tentacles are fully retracted, but the initial swims are relatively weak. The energy expended during swimming is the product of drag and velocity (Vogel, 1994). Energy can be conserved when drag is high by maintaining a low swim velocity but once the tentacles have been drawn into a more streamlined configuration, a greater velocity can be achieved with the same energy expenditure (Bone, 1981; Chain et al., 1981; Inoue et al., 2005).

In *Chelophyes* the action potentials recorded from the muscle epithelium become progressively longer during a swim sequence (Fig. 5F). Much of the Ca^2+^ necessary for contraction enters the muscle during the action potential plateau so that the longer the plateau, the stronger the contraction. At the same time K^+^ channels necessary for plateau termination enter an inactivated state. Recovery from this state is a slow process, and so fewer and fewer K^+^ channels remain available for repolarisation, and the action potentials get longer and longer (Inoue et al., 2005).

In contrast, when *Muggiaea* swims the contractions appear to be of uniform strength (see ‘Role of giant axons in siphonophores’, above). Analysis of voltage-clamp currents that flow across depolarised muscle membranes show that non-inactivating K^+^ channels help to terminate the action potential and make its duration less dependent on inactivating currents (R.W.M. and G. O. Mackie, unpublished). The resulting constant duration action potentials would account for *Muggiaea*’s uniform, peak instantaneous swimming velocity (Fig. 5D).

The Trachymedusae use similar tentacle management strategies; swimming is either delayed until the tentacles have been withdrawn, or an initial weak swim draws them into streamlined alignment. *Aglantha* has a large array of fine tentacles and how they are managed depends on whether it is undergoing fast or slow swimming. During fast swims, the tentacles are retracted immediately, the fast reaction time being achieved by the rapidly propagating impulses in the tentacle giant axons (conduction velocity 0.4–0.8 m s^−1^; Roberts and Mackie, 1980). Fig. 3C is a representation of the different neural circuits present in the nerve ring (Mackie and Meech, 1995).

During slow swimming, the tentacles contract progressively, and the swimming muscle response is initially weak (Fig. 4Cii). The strategy resembles that of *Chelophyes*, but the inactivating K^+^ channels are in the motor giant axons rather than the muscle (Meech and Mackie, 1993; Meech, 2015). Although the first axon spike in a slow swim series has an attenuated plateau and a marked undershoot, the K^+^ channels responsible inactivate quickly so that many later spikes are longer lasting, and the muscle responses are correspondingly greater (Fig. 4Cii).

Members of the Crossota clade of Trachymedusae also withdraw their tentacles only slowly (Meech et al., 2021). However, another near relative, *Colobonema*, swims with its tentacles extended (Fig. 2C) and its powerful fast swims may have evolved to compensate for the additional drag that that configuration imposes. Its slow swims may allow the animal to get up to speed, align its tentacles and maintain momentum.

## Discussion

According to Adrian Horridge (1977), a full account of animal behaviour calls for three layers of explanation. The first layer is ‘mechanistic reductionism’. This is purely descriptive and gives an account of interactions between neural components without explaining ‘why components and interactions are as they are’. Explaining why components and interactions ‘are as they are’ requires us to engage with the legacy of evolution, teasing out the ways that ‘selection has acted on what the ancestors have provided’. This second layer of explanation hints at the compromises of the past. The third and final level of explanation, what Horridge calls ‘mechanistic teleology’, confronts these compromises directly by examining how efficient the different components are in performing their functions.

Mechanistic teleology assumes that adaptive evolution has honed the components and their interactions into an optimised system. However, it is the system that is optimised, not the individual components. The individual components can participate in multiple different actions so that their nature and interactions are necessarily a compromise. In 1977, Horridge considered that explanations at level three were too complex to calculate, but that in the end no other approach would account for why the system is as it is.

Completing Horridge's programme would require careful comparative measurements of energy consumption – measurements that for the most part are yet to be done (but see the comparison between *Chelophyes* and *Abylopsis* by Bone and Trueman, 1982). If the primary function of slow swimming is food gathering, its comparative efficiency might be expressed as a function of the calorific value of the food gathered. However, this would conceal the fact that some species, like *Aequorea*, swim and feed continuously while others, like *Aglantha*, are intermittent swimmers and feeders. Placing a numerical value on the ability to escape swim is also a challenge, although it has been achieved for escape swims in crayfish (Herberholz et al., 2004).

Evaluations of the efficiency of physiological and behavioural processes in extant species should somehow take account of the flexibility required for survival in the face of what may be a variable physical environment. They should also recognise that the competition between species takes place amid a complex backdrop of ecological interdependence. The situation today is therefore not much different from that 45 years ago, and it is difficult to avoid the conclusion that such an analysis can be little more than a pipe dream unless the system selected is the simplest available. In the Cnidaria many of the simplest systems are neural or epithelial pathways concerned with escape, simplicity contributing to success by minimising response times.

If the value of the cost/benefit analysis required by Horridge's third level of explanation is unclear, teasing out the ways that ‘selection has acted on what the ancestors have provided’, the second level of explanation, is more promising. A behavioural asset like the ability to escape swim is certainly derived from pre-existing systems and components. Perhaps *Colobonema*’s strong swims evolved to overcome the drag imposed by a mass of extended tentacles. Or perhaps *Aglantha*’s strong swims were originally a way to flush damaging copepods from out of the swimming bell. In the Cnidaria damage, such as that inflicted by a copepod on the bell myoepithelium, is quickly repaired (Schmid et al., 1999) and if the repair process involves fusogen molecules, as it does in *Caenorhabditis elegans* (Neumann et al., 2015; Soulavie and Sundaram, 2016), it is easy to see how the mechanism might be co-opted for generating giant axons.

### Role of fusogens in axonal fusion

The anatomy of the giant axons in *Aglantha* suggests that they arise from the fusion of many smaller elements (see ‘Fusogens and giant axon formation’, above). The hypothesis proposed here is that the process depends on fusogen-like molecules. Fusogens are often small proteins that bring cell membranes together prior to cell fusion. Examples include the fusion-associated small transmembrane (FAST) protein that generates syncytia in fish cell lines infected with aquareovirus, the viral fusogen hemagglutinin and the *C. elegans* cell–cell fusogen proteins Epithelial Fusion Failure 1 (EFF-1) and Anchor-cell Fusion Failure 1 (AFF-1).

Fusogens are known to have a wide range of structures and their identification in the cnidarian genome is likely to be challenging. One starting point is to select regions of the genome coding for amphipathic helices. These are amino acid sequences that fold into a helical structure upon contact with a polar/non-polar interface and are known to be a characteristic feature of fusion proteins (Giménez-Andrés et al., 2018). Non-polar amino acids located every 3–4 residues along a polypeptide chain cause it to take up a α-helix formation with its interior face away from contact with water so that the fusogen adsorbs at lipid surfaces of cellular organelles. A short helical sequence could lie on the membrane surface and anchor the whole protein.

Hydrophobic moment plots can identify such sequences and distinguish between surface-seeking and transmembrane helical regions (Eisenberg et al., 1982). A web server (HeliQuest) is available to screen sequences with specific α-helical properties (Gautier et al., 2008). Keller (2011) has suggested that a HeliQuest-generated Eisenberg plot together with the HeliQuest lipid binding discrimination factor provide a good starting point to identify proteins likely to interact with lipid surfaces.

Many fusogen coding genes appear to be of retroviral origin. One example is Syncytin, which is the product of the envelope gene of a human endogenous retrovirus (Mi et al., 2000). Syncytin in its many forms is essential for the formation and growth of the placenta and its incorporation into the mammalian genome may have been a key step in the evolution of placental mammals. Screening human sequence databases for other viral envelope gene sequences has revealed other molecules with fusogen properties (Blaise et al., 2003) and this appears to be a useful model in the search for fusogens in the Cnidaria.

Knowing the lineage leading to a specific trait, it should be possible to identify the phylogenetic branch point, and then compare the genomes of species on either side of the node. How feasible it is to identify the genomic elements responsible for the trait will depend on many factors, such as the proportion of repetitive sequence (Stein et al., 2003). However, in *C. elegans* it has been possible to identify specific genes that have been repeatedly co-opted during the convergent evolution of, for example, self-fertility (Haag et al., 2018).

Identifying the fusogens responsible for giant axon fusion is just one step towards recognising other members of the developmental genetic toolkit. The work of Neumann et al. (2015) on axonal regeneration in *C. elegans*, showing changes in the subcellular location of the EFF-1 fusogen is an example of the kind of molecular steps involved. Other examples of axonal fusion are reviewed by Giordano-Santini et al. (2016) and Soulavie and Sundaram (2016).

### Molecular basis of behaviour

Attempts to deduce the building blocks of behaviour by comparing the genomes of closely related species are limited by reliance on extant species and the coarse temporal resolution that they provide. Moreover, there is the problem that these ‘snapshots’ of evolution have not been ‘fixed’ in time and the silver grains (or pixels) may have reformed themselves in highly misleading ways.

*Aglantha* and *Colobonema* are closely related but it is questionable whether their genomes are sufficiently close for comparisons to identify fine differences in molecular detail. In evolving giant axon-based escape swimming, *Aglantha* gained more than just the instructions for constructing giant axons; there are other refinements, such as a doubling of the signalling bandwidth. The low internal resistance of the giant axon not only made fast escape swim impulses possible, it also meant that impulses based on low amplitude currents could propagate below the escape swim threshold. As a result, slow swimming, instead of being limited to the bell margin, was displaced to the mid-bell (Meech, 2015).

It might be possible to highlight the key changes in giant axon evolution by comparing the genomes of species in which they have evolved independently. The forces driving such convergence may be either internal to the animal itself or connected to a complex web of environmental factors (Weibel, 1998). These constraints may be easier to separate in animals with a limited number of component parts. The deep sea Medusozoa, with their stable environment and simple body plan, are therefore an ideal platform to untangle the molecular origins of giant axon-based behaviour.
